# The CpxQ sRNA Negatively Regulates Skp To Prevent Mistargeting of β-Barrel Outer Membrane Proteins into the Cytoplasmic Membrane

**DOI:** 10.1128/mBio.00312-16

**Published:** 2016-04-05

**Authors:** Marcin Grabowicz, Daria Koren, Thomas J. Silhavy

**Affiliations:** Department of Molecular Biology, Princeton University, Princeton, New Jersey, USA

## Abstract

The promoter most strongly induced upon activation of the Cpx two-component envelope stress response is the *cpxP* promoter. The 3′ untranscribed region (UTR) of the *cpxP* transcript is shown to produce a small RNA (sRNA), CpxQ. We investigated the role of CpxQ in combating envelope stress. Remarkably, the two effectors specified by the transcript are deployed to combat distinct stresses in different cellular compartments. CpxP acts in both a regulatory negative-feedback loop and as an effector that combats periplasmic protein misfolding. We find that CpxQ combats toxicity at the inner membrane (IM) by downregulating the synthesis of the periplasmic chaperone Skp. Our data indicate that this regulation prevents Skp from inserting β-barrel outer membrane proteins (OMPs) into the IM, a lethal event that likely collapses the proton motive force. Our findings suggest that Skp can fold and directly insert OMPs into a lipid bilayer *in vivo* without the aid of the Bam complex.

## IMPORTANCE

Skp is a well-characterized periplasmic chaperone that binds unfolded OMPs. Surprisingly, we find that Skp can catalyze the folding and mistargeting of OMPs into the inner membrane without the aid of the other cellular proteins that normally assemble OMPs. Several OMPs function as diffusion pores. Accordingly, their mistargeting is lethal because it depolarizes the inner membrane. We show that the most highly expressed transcript of the Cpx stress response produces an sRNA from the 3′ UTR, CpxQ, which combats this potential toxicity by downregulating Skp production. Defects in OMP assembly trigger the σ^E^ response to upregulate factors, including Skp, that promote OMP folding. The Cpx response downregulates σ^E^. Our findings reveal that this heretofore puzzling hierarchy exists to protect the inner membrane.

## INTRODUCTION

Gram-negative bacteria build a complex envelope architecture where biologically distinct inner and outer membranes (IM and OM) are separated by an aqueous periplasmic space. Several macromolecular assembly machines are involved in envelope biogenesis, and some can function in the absence of available energy sources (1). The complexity of coordinate envelope biogenesis during cell growth is underpinned by several stress response systems that sense envelope defects and alter gene expression to either alleviate or clear the damage (2). Some of these systems are specific: for example, σ^E^ responds primarily to OM membrane defects (3, 4). On the other hand, the Cpx stress response is invoked to respond to stress signals originating throughout the envelope (5). The phage-shock-protein (Psp) response is activated by IM damage that reduces the proton motif force (PMF) (6).

At the core of the Cpx two-component stress response system are the sensor kinase CpxA and response regulator CpxR (5, 7). CpxA is a polytopic IM protein with dual kinase and phosphatase activity that can detect stress signals via its periplasmic sensing domain (8). Activation of CpxA leads to autophosphorylation and then phosphotransfer to CpxR, enabling it to alter transcription of regulon members (8, 9).

A set of mutations in *cpxA* cause constitutive activation of Cpx (8). These dominant cpxA* alleles include *cpxA17*, causing an A188E substitution proximal to the site of autophosphorylation; and *cpxA24*, resulting in a deletion within the periplasmic sensing domain. These mutations have proven valuable in identifying members of the CpxR regulon (10). One of the most highly upregulated genes is *cpxP*, located immediately upstream from *cpxRA*. CpxP is a periplasmic protein that completes a negative-feedback loop by inhibiting CpxA activation, likely by interacting with the sensing domain (9, 11, 12). However, CpxA* proteins are refractory to CpxP inhibition (13). A second highly upregulated CpxR target is *degP*, which encodes a periplasmic protein with dual chaperone and protease function (14).

cpxA* alleles suppress a variety of envelope toxicities, including a toxicity caused by tethering LamB to the IM by its uncleaved signal sequence (15), a jamming toxicity of the Sec machine with folded LamB-LacZ (15), a periplasmic toxicity that occurs when LamB-LacZ is fully translocated from the cytoplasm (15), and a distinct periplasmic toxicity caused by misfolded P-pilus subunits (16).

The *lamB*(*A23D*) mutation alters the signal peptidase cleavage site of the OM maltoporin and causes delayed release of the mature protein (17). Accordingly, LamB(A23D) is efficiently translocated into the periplasm through the Sec translocon but remains tethered to the IM via its signal peptide rather than being released and assembled into the OM (17). Expression of *lamB*(*A23D*) is induced by maltose, and the resultant high-level production is toxic. cpxA* alleles suppress this toxicity (15). DegP is required but not sufficient for suppression. It is not known why LamB(A23D) is toxic or which additional Cpx regulon members are required to suppress toxicity (15).

Jamming toxicity is caused by the LamB-LacZ42-1 fusion protein (18). The LamB sequence targets the fusion to the Sec translocon and initiates secretion; however, the LacZ sequence folds rapidly in the cytoplasm, and the folded fusion cannot pass through Sec. The fusion jams the translocon, prompting the FtsH protease to degrade SecY (19). cpxA* alleles suppress LamB-LacZ42-1 toxicity by inducing production of YccA, which acts to stabilize SecY against proteolysis, allowing time for the hybrid protein to clear the translocator (19).

LamB-LacZ jamming toxicity can also be relieved either by mutations in the signal sequence that allow cotranslational secretion (H*LamB-LacZ) or by mutations that prevent LacZ folding (LamB-LacZX90). However, efficient translocation of these fusions then causes a periplasmic toxicity because the cysteine-rich LacZ misfolds and aggregates in the oxidizing environment of the periplasm (20–22). cpxA* mutations fully suppress the toxicity of these fusions because they overexpress *degP*, and the periplasmic protease degrades the fusion proteins. In fact, heterologous overproduction of DegP is sufficient to suppress periplasmic LacZ toxicity (21).

The PapE and PapG subunits of the uropathogenic *Escherichia coli* P-pilus are chaperoned in the periplasm by PapD and brought to the PapC usher for assembly (23). In *E. coli* K-12, the absence of PapD causes pilin subunits to misfold, aggregate, and stimulate the Cpx stress response (24). CpxP acts as an adaptor that binds misfolded pilins and delivers them to DegP for proteolytic degradation along with CpxP itself (16, 25). Indeed, misfolded pilins sequester CpxP from CpxA to relieve inhibition and allow activation of the Cpx stress response (16, 24). Unlike periplasmic LacZ toxicity, resistance against PapE/G toxicity requires both *cpxP* and *degP* (16).

Recent work in *Salmonella* identified an Hfq-stabilized small RNA (sRNA) product, named CpxQ, that is derived from the 3′ untranscribed region (UTR) of the *cpxP* mRNA (26). Cpx inducing conditions strongly activate *cpxP* transcription and so increase production of CpxQ, suggesting this sRNA may play a direct role in response to stress. In this work, we assess the effect of CpxQ on the production of CpxP in *E. coli* and explore its contribution to combating the different stresses alleviated by Cpx. We show that production of CpxQ can lower the levels of CpxP. Moreover, we find that although CpxP and CpxQ originate from the same mRNA, they mature to combat unique stresses at different sites of the cell envelope.

## RESULTS

### CpxQ negatively regulates production of CpxP.

Since CpxQ and CpxP are products of the same mRNA transcript, it was possible the sRNA was produced at the expense of the transcript, causing lowered CpxP production (26). In wild-type cells, CpxP abundance is low and undetectable by immunoblotting. Hence, we employed a multicopy plasmid system to investigate any effect of CpxQ on CpxP. We used the previously described pCpxP plasmid that expresses *cpxP* from a heterologous *trc* promoter (13). The plasmid also encodes 44 bp of native 5′ *cpxP* sequence, including the native transcriptional start site but no sequence from the 3′ UTR region; transcription of *cpxP* is terminated by the plasmid-carried *rrnB* terminator.

**FIG 1:**
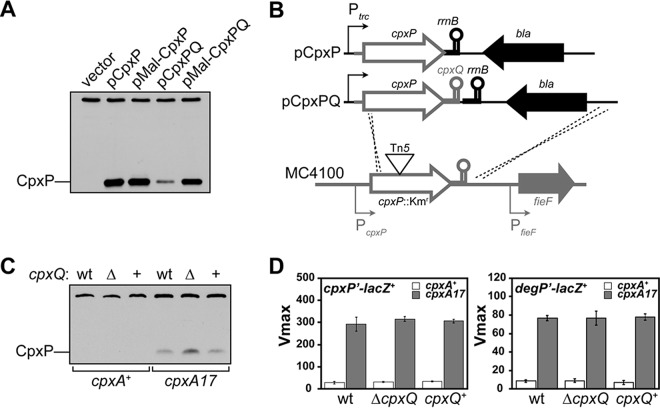
CpxQ reduces production of CpxP. (A) Anti-CpxP immunoblot of whole-cell CpxP levels in Δ*cpxP*::*kan* strains carrying multicopy plasmids. The upper band is a cross-reactive protein that serves as a loading control. (B) Recombination schematic for constructing Δ*cpxQ* and *cpxQ*^+^ strains at the native *cpxPQ* locus. (C) Anti-CpxP immunoblot of whole-cell CpxP levels produced from the *cpxPQ* wild-type locus (wt) or from *bla*-marked recombineered Δ*cpxQ* locus (Δ) and the isogenic control that contains *cpxQ* (+). The upper band is a cross-reactive protein that serves as a loading control. (D) Whole-cell β-galactosidase activity expressed from transcriptional LacZ reporters. The *cpxP* reporter is located away from the native locus and includes 410-nt upstream and 214-nt downstream sequences of the *cpxP* gene, relative to the translation start site. (*cpxQ* is not present in the reporter.) The data presented are means ± standard deviations from three experiments.

CpxP is abundant and readily detectable by immunoblotting in cells carrying pCpxP (Fig. 1A). We then created a derivative plasmid that introduced the entire 145-bp *cpxP* 3′ intergenic region (spanning CpxQ), creating pCpxPQ. The cellular levels of CpxP were markedly reduced by the presence of the *cpxQ* sequence (Fig. 1A). We concluded that *cpxQ* causes reduced production of CpxP.

We have been unable to predict likely sites of RNA-RNA interaction between CpxQ and the *cpxP* transcript. However, sRNA regulation often involves binding to the 5′ end of mRNA and can include binding to sequences that specify the signal peptide (27, 28). Therefore, we altered the 5′ end of the *cpxP* transcript by replacing the native signal peptide-encoding sequence, but not the ribosome-binding site, with one from MalE, creating plasmids pMal-CpxP and pMal-CpxPQ. Because the signal peptide is cleaved after translocation, the mature CpxP produced from each of the plasmids remained unaltered. The pMal-CpxP plasmid produced levels of CpxP that were comparable to those produced from pCpxP (Fig. 1A). Notably, a heterologous signal sequence region abrogated the negative effect of CpxQ on CpxP production, and CpxP levels from pMal-CpxPQ were much higher than from pCpxPQ and comparable to those from pCpxP (Fig. 1A). Each of the plasmids expressed comparable amounts of *cpxP* transcript, as determined by quantitative reverse transcription-PCR (qRT-PCR) (see Fig. S1 in the supplemental material). These results suggested that CpxQ reduces CpxP production in a manner that does not increase *cpxP* mRNA degradation and relies on the native 5′ mRNA sequence. Most likely, CpxQ exerts translational control over CpxP production.

### CpxQ lowers CpxP levels but does not alter CpxA activation.

The reduction in CpxP levels caused by CpxQ led us to consider whether this regulation contributes to the low levels of CpxP in wild-type cells by reducing mRNA or inhibiting *cpxP* mRNA translation directly or by some combination of direct and indirect effects. To address these questions under more physiological conditions, we recombineered the pCpxP and pCpxPQ constructs at the native *cpxPQ* locus to generate a Δ*cpxQ* deletion strain (pCpxP recombinant) and an isogenic control *cpxQ*^+^ strain (pCpxPQ recombinant). This recombination scheme preserved the native *cpxP* promoter and introduced a heterologous *rrnB* transcription terminator and a *bla* ampicillin resistance marker (Fig. 1B).

To monitor the indirect effect of CpxQ on CpxP-mediated CpxA activity, we used a *degP*′-*lacZ*^+^ transcriptional fusion that reports on the level of Cpx activation. CpxQ did not affect the extent of CpxA activation since we observed no changes in a *degP* expression from the *degP*′-*lacZ*^+^ reporter (Fig. 1D). Thus, the changes in CpxP levels are not the indirect result of changes in CpxA activity.

Consistent with previous reports, we were unable to detect CpxP in the wild-type *cpxA*^+^ background, but CpxP was easily detectable in the *cpxA17* background (16). The recombineered *cpxQ*^+^ strain produced CpxP levels equivalent to the wild type (Fig. 1C). However, we observed increased CpxP protein levels in the recombineered Δ*cpxQ* cells (Fig. 1C). This suggests that CpxQ downregulates *cpxP* directly.

We used a LacZ transcriptional reporter to determine whether CpxQ was acting directly to destabilize the *cpxP* mRNA. The *cpxP*′-*lacZ*^+^ transcriptional reporter is located at a heterologous site in the chromosome and measures mRNA levels expressed from the *cpxP* promoter. As noted above, the signal sequence coding region (nucleotides [nt] 1 to 63 of the *cpxP* gene) was required for CpxQ to lower CpxP levels. The *lacZ* reporter includes 410 nt of sequence upstream of *cpxP* as well as the first 214 nt of the *cpxP* gene (8). We observed that *cpxQ* had no effect on the amount of LacZ produced from the reporter either in *cpxA*^+^ or in *cpxA17* strains, suggesting that the abundance of the reporter mRNA is unchanged between strains (Fig. 1D). The results in this section confirm results obtained with plasmid constructs and demonstrate that CpxQ reduces CpxP levels by decreasing translation, not by destabilizing *cpxP* mRNA.

### CpxQ is not involved in combating misfolded pilin stress.

**FIG 2:**
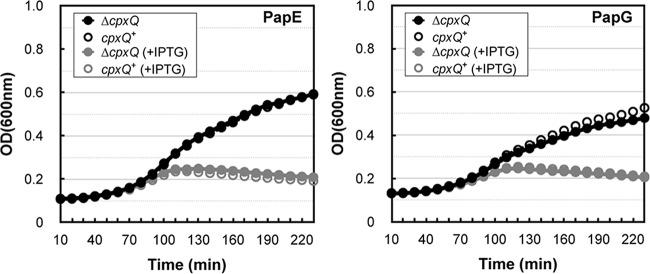
CpxQ does not combat misfolded pilin stress. Shown is growth of *cpxA*^+^ strains with either a deletion in *cpxQ* at the native chromosomal locus (Δ*cpxQ*) or an isogenic control locus that contains *cpxQ* (*cpxQ*^+^) during pilin subunit overexpression. The strains carry plasmids containing IPTG-inducible *papE* (pHJ13) and *papG* (pHJ8) and were cultured in media with (gray) or without (black) supplemented IPTG (10 µM).

The pilin subunits PapE and PapG are toxic when they are produced without their dedicated chaperone, PapD, and this periplasmic toxicity is suppressed by the cpxA* alleles. In this case, suppression requires Cpx regulon members DegP and CpxP (16). The increased production of CpxP that we observed in Δ*cpxQ* strains suggested that the sRNA could be involved in combating stress caused by misfolded pilin subunits. We envision two possibilities in a Δ*cpxQ* background: elevated CpxP levels could inhibit timely activation of CpxA to exacerbate toxicity, or alternatively, elevated CpxP levels could enhance clearance of misfolded pilins at the onset of stress. We constructed *cpxA*^+^ strains with or without *cpxQ* and carrying either pHJ8 or pHJ13 plasmids, which contain isopropyl-β-d-thiogalactopyranoside (IPTG)-inducible *papG* or *papE*, respectively. Strains were inoculated in media containing IPTG (10 µM) to overproduce PapE or PapG, and growth was monitored. We could not detect a difference in sensitivity to either misfolded PapE or PapG when *cpxQ* was deleted (Fig. 2). This result seemed consistent with the modest increase of CpxP levels in Δ*cpxQ* strains (Fig. 1C). Surprisingly, CpxQ regulation of CpxP levels does not affect the ability of cells to combat a periplasmic stress in which CpxP directly participates. What, then, is the physiological role of CpxQ?

### CpxQ contributes to combating the IM stress caused by tethered LamB.

**FIG 3:**
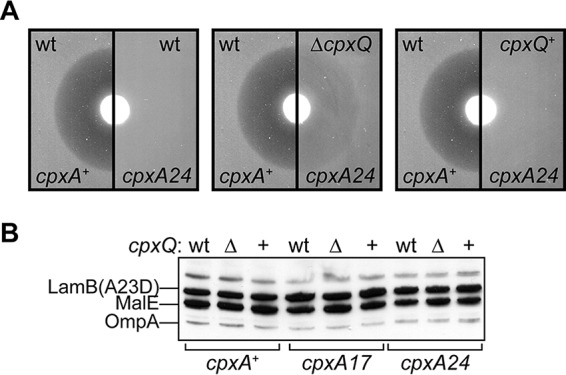
CpxQ is required for full suppression of *lamB*(*A23D*) by cpxA*. (A) Representative maltose disc diffusion assay images demonstrating zones of growth inhibition around a maltose disc. wt, native *cpxPQ* locus, compared to the deletion (Δ*cpxQ*) and its isogenic control (*cpxQ*^+^). (B) Combined anti-LamB and anti-MalE immunoblot of whole-cell levels of LamB(A23D) in cells grown in LB to mid-log phase and induced with 0.2% maltose for 60 min. Δ, *bla*-marked Δ*cpxQ* recombineered chromosomal deletion; +, the isogenic *bla*-marked recombineered control locus where *cpxQ* is present. OmpA is cross-reactive with anti-LamB and serves as a loading control. MalE serves as a control that measures the intensity of maltose induction.

Induction of *lamB*(*A23D*) expression by maltose is toxic and causes cell death. While cpxA* mutations suppress the toxicity of tethered LamB(A23D), neither the underlying mechanisms nor the Cpx regulon members involved are clear (17, 29). To investigate if CpxQ contributes to cpxA* suppression, we constructed isogenic Δ*cpxQ* and *cpxQ*^+^ strains in the *lamB*(*A23D*) background, with and without the cpxA* suppressor, and performed maltose disc diffusion assays to measure the zones of growth inhibition caused by the inducer. As expected, the *cpxA*^+^ strains were highly sensitive to inducer, and the absence of CpxQ sRNA did not alter maltose sensitivity (Table 1). However, cpxA* Δ*cpxQ* strains displayed an intermediate maltose sensitivity phenotype, while the control cpxA* *cpxQ*^+^ strains remained fully maltose resistant (Table 1). Specifically, we observed zones of growth inhibition in cpxA* Δ*cpxQ* strains that were the same size as those in *cpxA*^+^ strains, but with less cell death within the zone (Fig. 3A). We confirmed that this effect of CpxQ was not due to lowered production or increased degradation of LamB(A23D) following maltose induction (Fig. 3B).

**TABLE 1:** Maltose sensitivity profile of *lamB*(*A23D*) strains

Relevant strain background	Zone of inhibition (mm)^a^					
	*cpxA*^+^		*cpxA17*		*cpx24*	
	*cpxQ^+^*	Δ*cpx*Q	*cpxQ*^+^	Δ*cpxQ*	*cpxQ*^+^	Δ*cpxQ*
*lamB*(*A23D*)	21	20	0	(21)	0	(21)
*lamB*(*A23D*) pTrc99A	20	21	0	(21)	NT	NT
*lamB*(*A23D*) pCpxP	27	26	0	(20)	NT	NT

a The zone of inhibition is the diameter of growth clearance minus the 6-mm disc. Zones of inhibition with incomplete clearance are given within parentheses. NT, not tested.

### CpxQ does not alleviate LamB(A23D) toxicity by reducing CpxP.

Overproduction of CpxP is known to exacerbate *lamB*(*A23D*) maltose sensitivity in *cpxA*^+^ strains (13). We observed above that Δ*cpxQ* strains produce increased levels of CpxP in a *cpxA17* background. Perhaps this effect of CpxQ was responsible for increased maltose sensitivity in cpxA* Δ*cpxQ* strains? To test this, we introduced pCpxP and control pTrc99A plasmids into *lamB*(*A23D*) strains. The *cpxA*^+^ strains carrying pCpxP were indeed more maltose sensitive than the control strains (Table 1). However, in a *cpxA17* background, pCpxP did not alter maltose sensitivity phenotypes: the *cpxA17 cpxQ*^+^ strain remained fully resistant, while the *cpxA17* Δ*cpxQ* strain still displayed an intermediate maltose sensitivity (Table 1). Hence, elevated CpxP levels in cpxA* Δ*cpxQ* cells cannot account for the loss of suppression that occurs due to the absence of CpxQ. We conclude that cpxA*-mediated suppression of LamB(A23D) toxicity requires another CpxQ-regulated target.

### CpxQ is not required to combat toxicity caused by periplasmic LacZ.

Though it is tethered to the IM, the LamB(A23D) protein is localized in the periplasm. The periplasmic chaperone protease DegP contributes to, but is not sufficient for, cpxA* suppression of *lamB*(*A23D*) (15). As noted above, in the case of the periplasmic toxicity caused by misfolded P-pilus subunits, DegP is required for suppression, but the misfolded subunits must be presented to the protease by CpxP for degradation to occur (16). We wondered if CpxQ might regulate additional regulators of DegP activity. DegP overproduction is both necessary and sufficient for suppression of the H*LamB-LacZ or the LamB-LacZX90 fusion proteins that cause periplasmic stress. Hence, we used these fusions as sensitive reporters of DegP activity. We introduced Δ*cpxQ* or control *cpxQ*^+^ alleles to *cpxA*^+^ and cpxA* strains expressing each fusion. Measuring inducer sensitivity, we observed that Δ*cpxQ* had no effect on the maltose sensitivity profiles of either fusion in the sensitive *cpxA*^+^ background or in the suppressed cpxA* backgrounds (Table 2). These data suggest that CpxQ involvement in *lamB*(*A23D*) suppression does not occur by regulating DegP.

**TABLE 2:** Maltose sensitivity profile of *lamB-lacZ* fusion strains

Fusion	Zone of inhibition (mm)^a^					
	*cpxA*^+^		*cpxA17*		*cpx24*	
	*cpxQ*^+^	Δ*cpxQ*	*cpxQ*^+^	Δ*cpxQ*	*cpxQ*^+^	Δ*cpxQ*
*lamB-lacZX90*	26	25	0	0	0	0
H*lamB-lacZ	13	13	0	0	0	0
*lamB-lacZ42-1*	23	23	0	0	0	0

a The zone of inhibition is the diameter of growth clearance minus the 6-mm disc.

### CpxQ does not contribute to alleviating translocon jamming stress.

Jamming of the Sec translocon by the LamB-LacZ42-1 fusion protein is another stress that is suppressed by cpxA* alleles, and it is known that Cpx regulon member YccA contributes to this suppression by inhibiting the IM protease FtsH. To determine if these factors play a role in suppressing LamB(A23D), we constructed strains that expressed the LamB-LacZ42-1 fusion in both *cpxA*^+^ and cpxA* strains, each either lacking chromosomal *cpxQ* (Δ*cpxQ*) or with the control *cpxQ*^+^ locus. We then measured the maltose sensitivity of the strains. cpxA* alleles strongly suppress LamB-LacZ42-1 toxicity compared to *cpxA*^+^, but loss of *cpxQ* does not appreciably change the maltose sensitivity profile in either background (Table 2). We conclude that CpxQ is not involved in relieving jamming toxicity and that YccA and FtsH are not likely involved in the suppression of LamB(A23D).

### CpxQ combats LamB(A23D) IM stress by regulating the periplasmic chaperone Skp.

During the course of this work, Chao and Vogel had identified the cellular mRNA targets of CpxQ in *Salmonella* that had not been detected in microarray experiments (30). Notable among these targets was the Na^+^/H^+^ antiporter, NhaB, and the periplasmic chaperone Skp. Removing NhaB from any of the *lamB*(*A23D*) strains neither increased nor decreased toxicity regardless of the presence or absence of CpxQ (data not shown).

**FIG 4:**
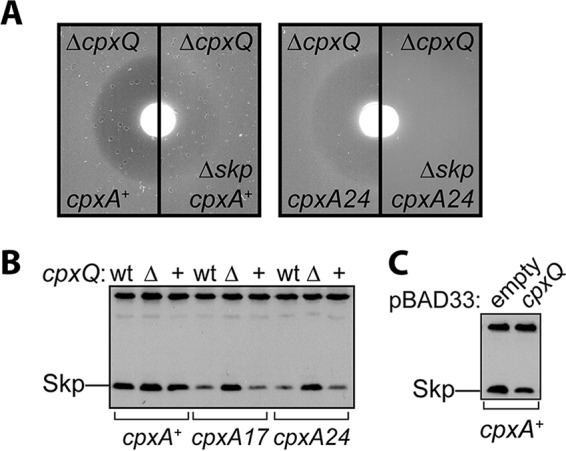
Loss of Skp suppresses LamB(A23D) toxicity and CpxQ acts by lowering Skp levels. (A) Representative maltose disc diffusion assay images demonstrating zones of growth inhibition around a disc containing maltose. (B) Immunoblotting of whole-cell Skp levels in *lamB*(*A23D*) strains. wt, wild-type *cpxPQ* locus; Δ, Δ*cpxQ* recombineered chromosomal deletion; +, isogenic recombineered control where *cpxQ* is present. (C) Immunoblotting of whole-cell Skp levels in *cpxA*^+^ plasmid carrying strains grown in the presence of 0.2% arabinose to induce expression of cloned *cpxQ*.

To determine if CpxQ-dependent regulation of Skp was required for alleviation of LamB(A23D) stress, we proceeded to delete *skp* from the *lamB*(*A23D*) strains. We observed that cpxA* *cpxQ*^+^ cells remained fully maltose resistant in the absence of Skp (Table 3). Strikingly, however, Δ*skp* restored full maltose resistance to cpxA* Δ*cpxQ* cells (Table 3; Fig. 4A) and also partially suppressed LamB(A23D) toxicity in *cpxA^+^* strains (Table 3; Fig. 4A). Importantly, the effect of removing Skp in alleviating LamB(A23D) toxicity was not simply due to the loss of a periplasmic chaperone: loss of *surA*, which encodes the major periplasmic chaperone for outer membrane proteins (OMPs), had no effect on the maltose sensitivity of *cpxA*^+^ cells or the resistance of cpxA* cells (data not shown).

**TABLE 3:** Maltose sensitivity profile of *lamB*(*A23D*) strains lacking Skp

Relevant strain background	Zone of inhibition (mm)^a^					
	*cpxA*^+^		*cpxA17*		*cpx24*	
	*cpxQ*^+^	Δ*cpxQ*	*cpxQ*^+^	Δ*cpxQ*	*cpxQ*^+^	Δ*cpxQ*
*lamB*(*A23D*)	21	20	0	(21)	0	(21)
*lamB*(*A23D*) Δ*skp*::*kan*	(18)	(18)	0	0	0	0

a The zone of inhibition is the diameter of growth clearance minus the 6-mm disc. Zones of inhibition with incomplete clearance are given within parentheses.

The fact that Δ*skp* suppressed maltose sensitivity demonstrated that Skp is involved in promoting LamB(A23D) toxicity. Furthermore, the finding that Δ*skp* restored maltose resistance to cpxA* Δ*cpxQ* cells strongly suggested that CpxQ contributes to combating LamB(A23D) toxicity by negatively regulating Skp. To test this hypothesis directly, we assessed Skp levels in *lamB*(*A23D*) strains by immunoblotting with anti-Skp antisera. In *cpxA*^+^ cells, Skp levels were abundant with or without *cpxQ* (Fig. 4B). On the other hand, the cpxA* mutations (where transcription from the *cpxPQ* promoter is strongly activated) resulted in markedly reduced Skp levels when *cpxQ* was present (Fig. 4B). In contrast, in cpxA* strains that lacked *cpxQ* Skp levels remained elevated and comparable to levels in *cpxA*^+^ strains (Fig. 4B). Furthermore, we were able to lower Skp levels in *cpxA*^+^ cells by overproducing CpxQ from an arabinose-inducible pBAD33 plasmid (Fig. 4C). Our data implicate Skp in contributing to the toxicity of LamB(A23D) and show that CpxQ negatively regulates Skp production when Cpx is activated.

### Skp promotes LamB(A23D)-mediated activation of the Psp response.

**FIG 5:**
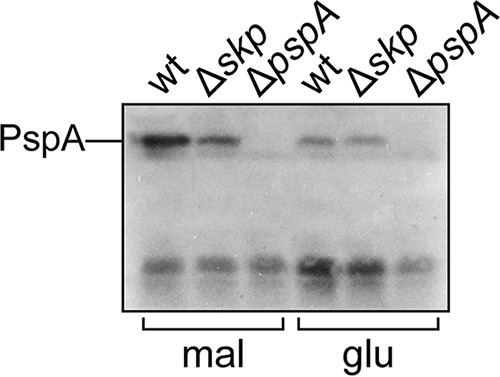
Skp is required for Psp activation in response to *lamB*(*A23D*) induction. Cultures of *cpxA^+^* lamB(*A23D*) cells with (wt) or without (Δ*skp*) *skp* were subcultured into media supplemented with either 0.2% maltose inducer (mal) or 0.2% glucose (glu) for 1 h. Cell lysates were then prepared and probed with anti-PspA antiserum.

In comparison with other examples of envelope toxicity, LamB(A23D) is unique in activating the Psp stress response; induction of *lamB*(*A23D*) causes elevated levels of PspA (17). Our data showed that Skp promotes LamB(A23D) toxicity, and introduction of Δ*skp* in *cpxA*^+^ cells partially suppresses inducer sensitivity. We wondered if this effect of Δ*skp* also lowered activation of the Psp response. We induced *lamB*(*A23D*) expression by growing *cpxA*^+^ cells in media supplemented with 0.2% maltose for 1 h and then assessed levels of PspA. As a control, we grew the same strains in media with 0.2% glucose to repress *lamB*(*A23D*) expression. We detected increased levels of PspA produced in *cpxA^+^ skp*^+^ cells treated with maltose, in agreement with prior observations (Fig. 5). In comparison, PspA levels remained low in *cpxA^+^* Δ*skp* cells (Fig. 5). We conclude that Skp promotes LamB(A23D) toxicity in a manner that activates the Psp response.

## DISCUSSION

Despite being produced from the same mRNA, our results show that CpxP protein and CpxQ sRNA mature to become effectors that combat distinct stresses in different compartments of the cell envelope. CpxP functions to alleviate P-pilus misfolding in the periplasm, but CpxQ combats stress at the IM modeled by the LamB(A23D) mutant protein. The *lamB*(*A23D*) mutation alters the signal peptidase cleavage site such that the translocated protein remains tethered to the IM by its uncleaved signal peptide. When production of LamB(A23D) is increased by maltose addition, toxicity is apparent. Importantly, toxicity requires production of the full-length LamB(A23D) protein. Amber mutations that cause C-terminal truncation of LamB abolish its ability to fold; when such mutations are combined with *lamB*(*A23D*), they fully relieve toxicity (17). Clearly, then, toxicity requires the C-terminal region either (i) because it is itself the direct cause of toxicity or (ii) because it is required for LamB(A23D) to remain folding competent. In support of the latter model, our findings demonstrate that the pro-folding factor Skp promotes toxicity. Hence, induction of the Psp response and toxicity likely arises from a Skp-dependent folding of tethered LamB(A23D) into the IM.

We suggest that Skp-dependent folding and insertion of LamB(A23D) into the IM creates an ion-conducting pore in the IM that disrupts the PMF. In support of this hypothesis, the Psp stress response, which is known to be activated by conditions that collapse the proton gradient across the IM (6, 31), is strongly stimulated by LamB(A23D) production (17). Indeed, filamentous phage produce pore-forming proteins that deplete PMF by this mechanism, and these are well-characterized inducers of the Psp response (6). Moreover, translocation-defective mutant OMPs or secretins lacking their pilotin also induce the Psp responses; in cells lacking the major effector PspA, these proteins are known to deplete PMF and cause toxicity (31–33). Since it is tethered to the IM, LamB(A23D) is perhaps uniquely potent in causing toxicity even in *pspA*^+^ cells.

It is clear that OMPs inherently possess the requisite structural information to fold and insert directly into membranes *in vitro*, provided that aggregation is prevented by denaturants such as urea (34). *In vivo*, following translocation, periplasmic chaperones such as SurA and Skp prevent the hydrophobic OMPs from aggregation and maintain them in a folding-competent state. From the periplasm, OMPs could fold and insert directly into the IM or the OM since either bilayer likely imposes the same energetic barrier (35). However, direct OMP folding and membrane insertion are slow; *in vivo* the BamABCDE complex within the OM lowers the kinetic barrier for OMP assembly and catalyzes folding and insertion into the correct membrane (35, 36).

In *E. coli*, SurA is the major chaperone for OMPs. Indeed, loss of SurA causes severe defects in OMP assembly, and cells survive the loss only because they are rescued by strong induction of the σ^E^ stress response. In contrast, there is no OMP that prefers Skp over SurA, and loss of Skp causes no OMP assembly defects and does not result in stress response induction. Skp does play a redundant role with FkpA in the assembly of LptD (37), but it functions primarily to rescue OMPs that have fallen off the normal assembly pathway (38). Since loss of Skp prevents folding and insertion of LamB(A23D) into the IM, we must conclude that this is a function that Skp does not share with SurA.

The fact that Skp can insert LamB(A23D) into the IM and SurA cannot is consistent with several experimental observations made previously. Most strikingly, Skp differs from SurA by its ability to promote OMP folding and insertion *in vitro*. SurA maintains OMPs in a folding-competent state, but membrane insertion requires the Bam complex (39–41). Skp, on the other hand, functions as a homotrimer with a large central cavity that can accommodate an entire unfolded OMP (42). Skp allows bound unfolded OMPs to undergo rapid conformational shifts, and it is sufficient to catalyze OMP insertion into membranes directly *in vitro* (41, 43–45).

Our findings indicate that OMPs can be assembled into membranes by Skp *in vivo* without participation from the OM Bam complex. In particular, our data suggest that Skp can assemble a β-barrel protein like LamB(A23D) that is tethered to the IM into the bilayer. Because high-level production of IM-tethered LamB is required to induce toxicity, it is likely that Skp cannot do this efficiently. We can detect it because only a few pores are required to inhibit cell growth. We do think it likely that Skp can assemble proteins directly into the OM as well. At present, we do not have an *in vivo* assay sensitive enough to detect this, but such an activity could rescue cells with defects in normal OMP biogenesis for one reason or another.

The Cpx response is an envelope stress response, and it seems counterintuitive that it would seek to lower Skp levels—why reduce the abundance of a chaperone that prevents aggregation of misfolded OMPs? We believe the *râison d’être* of such regulation is more apparent when considered in the context of mounting evidence that the primary responsibility of the Cpx response is to maintain IM homeostasis (5). Conditions that impede efficient OMP assembly into the OM trigger the σ^E^ response to promote recovery of OMP folding and assembly by overproducing the Bam complex and chaperones, including SurA and Skp (46). However, our findings suggest that Skp-OMP interactions can result in toxic OMP folding into the IM, and this mistargeting likely increases if Bam function is compromised. We suggest that this presents a challenge met by the Cpx response. To protect the IM, the Cpx response directly downregulates the abundant OMPs (47, 48) and negatively regulates the σ^E^ response (49), and as we show here, the Cpx response directly reduces the production of Skp via the CpxQ sRNA. It is tempting to think that this Cpx-σ^E^ cross-regulatory axis is designed to allow an initial attempt at recovery from OMP stress, which can then overridden by Cpx seeking to protect IM integrity so that energy generation can continue. Notably, a Δ*cpxR* mutation causes conditional lethality in strains where OMP biogenesis is compromised (47).

Chao and Vogel demonstrate in *Salmonella* that CpxQ represents the first bacterial *trans*-acting global regulatory sRNA that is produced from the 3′ UTR of an mRNA (30). We have no reason to believe that biogenesis of CpxQ differs in *E. coli*. However, we did observe that Δ*cpxQ* results in higher levels of CpxP in *E. coli*, an effect not seen in *Salmonella*. Apparently there are differences in sRNA regulation of the Cpx regulon between *E. coli* and *Salmonella*. This is not uncommon; an instructive example is the loss of the RNA chaperone Hfq, which triggers activation of Cpx responses in enteropathogenic *E. coli* strains but has no effect in *E. coli* K-12 strains (50). Our data suggest that the 5′ sequence of the *cpxPQ* mRNA is required for CpxQ-mediated regulation of CpxP production. However, the physiological significance of this regulation is not immediately clear. In fact, in response to pilin misfolding, a stress condition known to require CpxP, we show that Δ*cpxQ* has no effect. More work is required to understand why *E. coli* strives to keep the levels of CpxP so low.

## MATERIALS AND METHODS

### Bacterial strains, plasmids, and growth conditions.

All strains and plasmids used in this study are listed in Table S1 in the supplemental material. Strains were routinely maintained in Luria medium (Miller), except for the *lamB*-*lacZ* and *lamB*(*A23D*) strains, which were maintained in M63 minimal medium supplemented with 0.2% (wt/vol) glucose at all times. cpxA* strains were maintained at 30°C in the presence of amikacin (1.5 µg/ml). The Δ*skp*::*kan* allele was obtained from the Keio collection (51).

### Plasmid construction.

All oligonucleotides used in this study are listed in Table S2 in the supplemental material. The plasmid pCpxP was constructed by cloning *cpxP* amplified with primers cpxP5′*Eco* and mg_pCpxP into the EcoRI and HindIII sites of pTrc99A (13). The *cpxP* 3′ UTR was cloned by amplifying *cpxPQ* with primers CpxP_intF_BglII and cpxP_3_HindIII and cloning the product into the BglII and HindIII sites of pCpxP. The *malE* signal sequence was amplified with primers OE_MalE_F and ssMalE_R and cloned by overlap-extension PCR with pMG95. The *mal*-*cpxP* construct was then subcloned from the pMG95 derivative, using EcoRI and HindIII, into the same sites of pTrc99A, creating pMal-CpxP.

### Chromosomal *cpxPQ* constructs.

To facilitate recombineering, a pCpxPQ equivalent plasmid was constructed with a shorter 3′ UTR region (to the promoter of *fieF*) that was cloned from an amplicon of cpxP5′*Eco* and cpxP_3s_HindIII.

To generate double-stranded DNA (dsDNA) for recombineering, the *cpxPQ* locus was PCR amplified from pCpxP and pCpxPQ with primers cpxP_5 and primer cpxP_3. The PCR products were used to transform strain DK10 (DY378 Δ*cpxP*::*kan*), selecting for Amp^r^ transformants and then screening for Kan^s^. Successful recombination generated *cpxP*::*rrnB*-*bla* and *cpxPQ*::*rrnB*-*bla* alleles. Kanamycin-resistant alleles were generated by targeting the *bla* gene for a second recombination when the constructed strains were transformed with dsDNA from an amplification of pKD4 (52) with primers Chr_Amp2Kan_p1 and Chr_Amp2Kan_p2. Recombinants were selected for Kan^r^ and screened for Amp^s^.

Both Amp^r^ and Kan^r^ alleles were moved routinely by P1*vir* transduction. Because the *cpxA* and *cpxP* loci are tightly linked, to facilitate strain construction involving cpxA* alleles, the Amp^r^ or Kan^r^ constructs were first linked to a *cpxA*::*cam* allele for to cotransduction. Transductants of cpxA* strains were selected for Amp^r^/Kan^r^ and then screened for Cam^s^.

### β-Galactosidase assays.

Overnight cultures subcultured 1:50 into LB. Cultures were grown for 1 to 2 h to mid-log phase. Equivalent *A*_600_ cell densities were taken, pelleted, and permeabilized using chloroform and SDS. Measurement of β-galactosidase activity for *ortho*-nitrophenyl-β-d-galactopyranoside (ONPG) hydrolysis was performed in triplicate (53), measuring spectrophotometric readings each minute during a 15-min time course, and the *V*_max_ was calculated.

### Maltose disc diffusion assay.

Overnight cultures were mixed with 3 ml of molten M63 medium top agar (agar at 0.75% wt/vol) supplemented with 0.2% (wt/vol) glycerol, spread onto a plate of M63 glycerol (agar at 1.5% wt/vol), and allowed to solidify. Filter discs infused with 10 ml of 20% maltose were placed in the center to the plate. Plates were incubated upright at 30°C overnight, and the diameters of zones of growth inhibition were measured.

### Immunoblotting.

Cultures were grown to mid-log phase, and samples were standardized by *A*_600_. Aliquots were taken, pelleted, and resuspended in Laemmli buffer. Samples were first boiled and then resolved by SDS-PAGE before being transferred to nitrocellulose membranes. Immobilized samples were probed with polyclonal rabbit anti-CpxP (1:5,000), anti-LamB (1:30,000), or anti-Skp (1:8,000) antisera or anti-PspA (raised against the *Yersinia enterocolitica* protein [1:5,000]) as indicated. Membranes were subsequently washed, incubated with donkey anti-rabbit secondary antibody conjugated to horseradish peroxidase (used at 1:10,000), and developed with enhanced chemiluminescence substrate (Amersham). Blots were visualized by exposure to X-ray film (Denville).

## SUPPLEMENTAL MATERIAL

Figure S1  Relative abundance of *cpxP* mRNA produced from plasmid constructs. Plasmid-carrying Δ*cpxP*::*kan* strains were grown to the mid-log phase. Total RNA was isolated (RNeasy; Qiagen) and used to prepare cDNA. qRT-PCR was performed using primers cpxP_qRT_F (5′ CTCCTGTTAATGTTAGCGAACTGG 3′) and cpxP_qRT_R (5′ ATTCGTTGTTGATGTTTCTCGTTT 3′), which amplify a 208-bp product within the *cpxP* open reading frame (ORF). Fold change was calculated by the threshold cycle (ΔΔ*C_T_*) method. Data represent two biological replicates. Download Figure S1, PDF file, 0.1 MB

Table S1  Strains and plasmids used in this study. Table S1, PDF file, 0.2 MB

Table S2  Oligonucleotides used in this study. Table S2, PDF file, 0.1 MB
