# Supplementary material for: The CpxQ sRNA Negatively Regulates Skp To Prevent Mistargeting of β-Barrel Outer Membrane Proteins into the Cytoplasmic Membrane
Source: mBio. 2016 Apr 5;7(2):e00312-16. doi: 10.1128/mBio.00312-16 (PMC4817254; doi:10.1128/mBio.00312-16)
Supplement: Figure S1 — Relative abundance of cpxP mRNA produced from plasmid constructs. Plasmid-carrying ΔcpxP::kan strains were grown to the mid-log phase. Total RNA was isolated (RNeasy; Qiagen) and used to prepare cDNA. qRT-PCR was performed using primers cpxP_qRT_F (5′ CTCCTGTTAATGTTAGCGAACTGG 3′) and cpxP_qRT_R (5′ ATTCGTTGTTGATGTTTCTCGTTT 3′), which amplify a 208-bp product within the cpxP open reading frame (ORF). Fold change was calculated by the threshold cycle (ΔΔCT) method. Data represent two biological replicates. Download [file mbo002162753sf1.pdf]

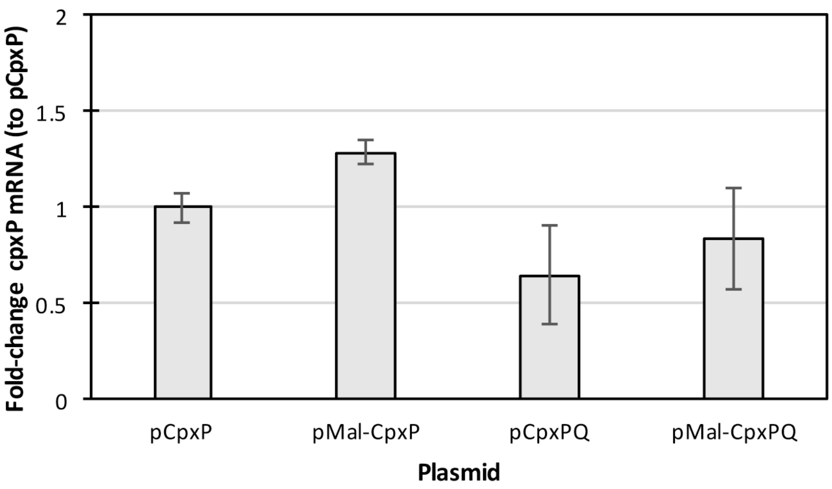

Figure S1. Relative abundance of *cpxP* mRNA produced from plasmid constructs. Plasmid carrying Δ*cpxP*::*kan* strains were grown to mid-log phase. Total RNA was isolated (RNeasy, Qiagen) and used to prepare cDNA. qRT-PCR was performed using primers cpxP_qRT_F (5'-ctcctgttaatgttagcgaactgg-3') and cpxP_qRT_R (5'-attcgttgttgatgtttctcgttt-3') that amplify a 208 bp product within the *cpxP* ORF. Fold-change was calculated by the $\Delta\Delta$Ct method. Data represent two biological replicates.
