# Supplementary material for: The CpxQ sRNA Negatively Regulates Skp To Prevent Mistargeting of β-Barrel Outer Membrane Proteins into the Cytoplasmic Membrane
Source: mBio. 2016 Apr 5;7(2):e00312-16. doi: 10.1128/mBio.00312-16 (PMC4817254; doi:10.1128/mBio.00312-16)
Supplement: Table S1 — Strains and plasmids used in this study. [file mbo002162753st1.pdf]

Table S1 Strains and plasmids used in this study

Strain or plasmids	Genotype or description	Reference or source
MC4100	F^- *araD139* Δ(*arg-lac*)*U169 rpsL150 relA1 flbB5301 deoC1 ptsF25 thi*	(Casadaban, 1976)
NR754	MC4100 Ara+	(Button et al., 2007)
MG2298	NR754 *cpxP*::*rrnB-bla* / pHJ13	This study
MG2299	NR754 *cpxPQ*::*rrnB-bla* / pHJ13	This study
MG2587	NR754 *cpxP*::*rrnB-bla* / pHJ8	This study
MG2588	NR754 *cpxPQ*::*rrnB-bla* / pHJ8	This study
MG2582	NR754 *ΔcpxP*::*kan* / pTrc99A	This study
MG2583	NR754 *ΔcpxP*::*kan* / pCpxP	This study
MG2584	NR754 *ΔcpxP*::*kan* / pMal-CpxP	This study
MG2585	NR754 *ΔcpxP*::*kan* / pCpxPQ	This study
MG2586	NR754 *ΔcpxP*::*kan* / pMal-CpxPQ	This study
DY378	W3110 λcI857 Δ(cro-bioA)	(Yu et al., 2000)
DK10	DY378 Δ*cpxP*::*kan*	This study
**Transcriptional LacZ reporter strains**		
TR50	MC4100 λRS88[*cpxP'-lacZ*$^+$]	(Raivio and Silhavy, 1997)
MG2404	MC4100 λRS88[*cpxP'-lacZ*$^+$] *cpxP*::*rrnB-kan*	This study
MG2405	MC4100 λRS88[*cpxP'-lacZ*$^+$] *cpxPQ*::*rrnB-kan*	This study
MG2399	MC4100 λRS88[*cpxP'-lacZ*$^+$] *cpxA17*	This study
MG2410	MC4100 λRS88[*cpxP'-lacZ*$^+$] *cpxA17 cpxP*::*rrnB-kan*	This study
MG2411	MC4100 λRS88[*cpxP'-lacZ*$^+$] *cpxA17 cpxPQ*::*rrnB-kan*	This study
TR50	MC4100 λRS88[*degP'-lacZ*$^+$]	(Raivio and Silhavy, 1997)
MG2406	MC4100 λRS88[*degP'-lacZ*$^+$] *cpxP*::*rrnB-kan*	This study
MG2407	MC4100 λRS88[*degP'-lacZ*$^+$] *cpxPQ*::*rrnB-kan*	This study
MG2398	MC4100 λRS88[*degP'-lacZ*$^+$] *cpxA17*	This study
MG2408	MC4100 λRS88[*degP'-lacZ*$^+$] *cpxA17 cpxP*::*rrnB-kan*	This study
MG2409	MC4100 λRS88[*degP'-lacZ*$^+$] *cpxA17 cpxPQ*::*rrnB-kan*	This study
***lamBA23D* strains**		

JHC285	MC4100 *zjb*::Tn*10 lamBA23D* (*cpxQ*^{wt})	(Carlson and Silhavy, 1993)
MG2416	JHC285 *cpxP*::*rrnB-bla* (Δ*cpxQ*)	This study
MG2417	JHC285 *cpxPQ*::*rrnB-bla* (*cpxQ*$^{+}$)	This study
MG2418	JHC285 *cpxA17* (*cpxQ*^{wt})	This study
MG2419	JHC285 *cpxA17 cpxP*::*rrnB-bla* (Δ*cpxQ*)	This study
MG2420	JHC285 *cpxA17 cpxPQ*::*rrnB-bla* (*cpxQ*$^{+}$)	This study
MG2421	JHC285 *cpxA24* (*cpxQ*^{wt})	This study
MG2422	JHC285 *cpxA24 cpxP*::*rrnB-bla* (Δ*cpxQ*)	This study
MG2423	JHC285 *cpxA24 cpxPQ*::*rrnB-bla* (*cpxQ*$^{+}$)	This study
MG2607	JHC285 / pTrc99A	This study
MG2608	JHC285 / pCpxP	This study
MG2713	MG2416 / pTrc99A	This study
MG2630	MG2416 / pCpxP	This study
MG2714	MG2417 / pTrc99A	This study
MG2631	MG2417 / pCpxP	This study
MG2415	MG2419 / pTrc99A	This study
MG2631	MG2419 / pCpxP	This study
MG2716	MG2420 / pTrc99A	This study
MG2633	MG2420 / pCpxP	This study
MG2634	MG2416 Δ*skp*::*kan*	This study
MG2635	MG2417 Δ*skp*::*kan*	This study
MG2637	MG2419 Δ*skp*::*kan*	This study
MG2638	MG2420 Δ*skp*::*kan*	This study
MG2640	MG2422 Δ*skp*::*kan*	This study
MG2641	MG2423 Δ*skp*::*kan*	This study
lamB-lacZ42-1 **strains**		
JCM912	MC4100 Φ(*lamB-lacZ*)hyb42-1 *cpxA*⁺	(Dwyer et al., 2014)
WBS226	MC4100 Φ(lamB-lacZ) hyb42-1 *cpxA17*	(Cosma et al., 1995)
NJH187	MC4100 Φ(lamB-lacZ) hyb42-1 cpxA24	(Cosma et al., 1995)
MG2566	JCM912 *cpxA*$^{+}$ *cpxP*::*rrnB-kan* (Δ*cpxQ*)	This study
MG2567	JCM912 *cpxA*$^{+}$*cpxPQ*::*rrnB-kan* (*cpxQ*$^{+}$)	This study
MG2568	WBS226 *cpxA17 cpxP*::*rrnB-kan* (Δ*cpxQ*)	This study
MG2569	WBS226 *cpxA17cpxPQ*::*rrnB-kana* (*cpxQ*$^{+}$)	This study
MG2570	NJH187 *cpxA24 cpxP*::*rrnB-kan* (Δ*cpxQ*)	This study

MG2571	NJH187 *cpxA24 cpxPQ*::*rrnB-kan* (*cpxQ*$^{+}$)	This study
***H*lamB-lacZ* strains**		
JCM944	MC4100 Ara⁺ ϕ(*H*lamB'-'lacZ*) Hyb42-1 [λp1(209)]	(Dwyer et al., 2014)
MG2673	JCM944 *cpxA*$^{+}$ *cpxP*::*rrnB-bla* (Δ*cpxQ*)	This study
MG2674	JCM944 *cpxA*$^{+}$ *cpxPQ*::*rrnB-bla* (*cpxQ*$^{+}$)	This study
MG2614	JCM944 *cpxA17 cpxP*::*rrnB-bla* (Δ*cpxQ*)	This study
MG2615	JCM944 *cpxA17 cpxPQ*::*rrnB-bla* (*cpxQ*$^{+}$)	This study
MG2616	JCM944 *cpxA24 cpxP*::*rrnB-bla* (Δ*cpxQ*)	This study
MG2617	JCM944 *cpxA24 cpxPQ*::*rrnB-bla* (*cpxQ*$^{+}$)	This study
***lamB-lacZX90* strains**		
JCM914	MC4100 Ara⁺ ϕ(*lamB'-'lacZX90*) Hyb42-1 [λp1(209)]	(Dwyer et al., 2014)
MG2384	JCM914 *cpxA*$^{+}$ *cpxP*::*rrnB-bla* (Δ*cpxQ*)	This study
MG2385	JCM914 *cpxA*$^{+}$ *cpxPQ*::*rrnB-bla* (*cpxQ*$^{+}$)	This study
MG2386	JCM914 *cpxA17 cpxP*::*rrnB-bla* (Δ*cpxQ*)	This study
MG2387	JCM914 *cpxA17 cpxPQ*::*rrnB-bla* (*cpxQ*$^{+}$)	This study
MG2388	JCM914 *cpxA24 cpxP*::*rrnB-bla* (Δ*cpxQ*)	This study
MG2389	JCM914 *cpxA24 cpxPQ*::*rrnB-bla* (*cpxQ*$^{+}$)	This study
**Plasmids**		
pTrc99A	Cloning vector, pBR322 *ori*, Amp^{R}	Lab stocks
pCpxP	*cpxP* cloned into *Eco*RI-*Hind*III of pTrc99A, Amp^{R}	(Raivio et al., 1999)
pMal-CpxP	*malE-cpxP* cloned into *Eco*RI-*Hind*III of pTrc99A,Amp^{R}	This study
pCpxPQ	*cpxPQ* with entire 3' intergenic region cloned into *Eco*RI-*Hind*III of pTrc99A, Amp^{R}	This study
pMal-CpxPQ	*malE-cpxPQ* with entire 3' intergenic region cloned into *Eco*RI-*Hind*III of pTrc99A, Amp^{R}	This study
pMal-CpxPQshort	*malE-cpxPQ* with shortened 3'UTR cloned into *Eco*RI-*Hind*III of pTrc99A, Amp^{R}	This study
pBAD33	Cloning vector, pACYC *ori*, Cam^{R}	(Guzman et al., 1995)
pBAD33*cpxQ*	*cpxQ* cloned at the transcriptional start site downstream from P_{araBAD} with primers pBAD_sRNA_F and pBAD_sRNA_R	This study
