# Supplementary material for: The CpxQ sRNA Negatively Regulates Skp To Prevent Mistargeting of β-Barrel Outer Membrane Proteins into the Cytoplasmic Membrane
Source: mBio. 2016 Apr 5;7(2):e00312-16. doi: 10.1128/mBio.00312-16 (PMC4817254; doi:10.1128/mBio.00312-16)
Supplement: Table S2 — Oligonucleotides used in this study. [file mbo002162753st2.pdf]

Table S2 Oligonucleotides used in this study

**Name**	**Sequence (5’ – 3’)**
cpxP_5	TCTCTATCGTTGAATCGCGACA
cpxP_3	CGCTAGTATAACGGAAGCAAATCATCTGCAATGCATTAAGCAGCAGGCAAGTTTTTTTGTTTGCAAGCAGCA
cpxP_3_HindIII	TCAAGCTTAAATACTCCCGCTATCAACTGAC
cpxP_3s_HindIII	ATCAAGCTTGAGGATAAAAAAAACCCCCAC
cpxP_intF_BglII	ATGCGAGATCTTATGCAACAG
cpxP5’Eco	GGAATTCCCTCTCTATCGTTGAATCGCG
mg_pCpxP	GCAAGGAAAACAAGCTTTACTACTGG
OE_MalE_F	GACAGAAAGATTTTGGGAGCAAATGATGAAAATAAAAACAGGTGCACGCATCC
ssMalE_R	CCAGTTATCGCCTGAACCGACTTCAGCGGCGAGAGCCGAGGCGGAAAACATC
Chr_Amp2Kan_p1	TCAAATTAAGCAGAAGGCCATCCTGACGGATGGCCTTTTTGCGTTTCTACAAACTCTTTTTGTTTATTTTTCTAAATACAGTGTAGGCTGGAGCTGCTTC
Chr_Amp2Kan_p2	TGATTCATAAATACTCCCGCTATCAACTGACGCTAGTATAACGGAAGCAAATCATCTGCAATGCATTAAGCAGCAGGCAAATGGGAATTAGCCATGGTCC
pBAD_sRNA_F	GTTTTCCTTGCCATAGACACCATCCCTGTCTTCCCCCACATGCTGTGGGGGTTTTTTTACAGATTAAATCAGAACGCAGAAGC
pBAD_sRNA_R	TGGAGAAACAGTAGAGAGTTGCGATA
cpxP_qRT_F	CTCCTGTTAATGTTAGCGAACTGG
cpxP_qRT_R	ATTCGTTGTTGATGTTTCTCGTTT
